# Supplementary material for: A Novel Method for the Background Signal Correction in SP-ICP-MS Analysis of the Sizes of Titanium Dioxide Nanoparticles in Cosmetic Samples
Source: Molecules. 2022 Nov 10;27(22):7748. doi: 10.3390/molecules27227748 (PMC9692317; doi:10.3390/molecules27227748)
Supplement: Supplementary file 1 [file molecules-27-07748-s001.zip › molecules-1992635-supplementary.pdf]

A novel method for the background signal correction in SP-ICP-MS analysis of the sizes of titanium dioxide nanoparticles in cosmetic samples

Zaual A. Temerdashev [1,*], Olga A. Galitskaya [1] and Mikhail A. Bolshov [2]

[1] Kuban State University, Faculty of Chemistry and High Technologies, Krasnodar, 350040 Russia
[2] Institute of Spectroscopy, Russian Academy of Sciences, Troitsk, Moscow, 108840 Russia
[*] Correspondence: temza@kubsu.ru

Supplementary information

Table S1. Signal intensity change at m/z 48 with sequential dilution of cosmetic product samples.

Dilution factor	Intensity, counts	
	sample 1	sample 2
1	102	46
10	46	16
100	19	**15** [a]
1000	**15** [a]	**15** [a]

[a] corresponds to the intensity of the background signal

Table S2. Change in the characteristics of the background signal in the time-resolved mode in the dwell time range of 0.1-20 ms.

**Dwell time (ms)**		**0.1**	**0.3**	**0.5**	**0.7**	**0.9**	**1**	**3**	**5**	**7**	**9**	**10**	**12**	**14**	**16**	**18**	**20**
Intensity range (counts)		0-5	0-8	0-11	0-13	0-14	0-16	3-29	10-42	17-53	24-64	28-70	39-85	44-94	52-105	60-117	65-124
Range width (counts)		6	9	12	14	15	17	27	33	37	41	43	47	51	54	58	60
Zero-intensity windows (%)		68.0	26.8	10.4	4.0	1.4	0.9	0	0	0	0	0	0	0	0	0	0
Intensity of the maximum distribution (counts)		0	1	2	3	4	4	14	25	32	44	49	58	68	79	89	98
Medium intensity (counts)		1	2	3	3	4	5	14	24	33	43	48	60	68	77	86	95
**σ** (counts)		0.5	1.0	1.4	1.8	2.0	2.2	3.8	5.1	5.9	7.0	7.4	8.8	9.4	9.9	10.6	11.0
Background signal threshold (counts)	3σ	3	5	7	9	11	12	26	39	50	64	70	86	97	107	118	128
	5σ	4	7	9	12	15	16	33	50	62	78	85	104	115	126	139	150
	10σ	6	11	16	21	25	27	52	75	92	113	122	148	162	176	192	205

Table S3. The change in the background signal (LOD) and the number of TiO_2 NPs calculated by the SPCal software [1] at the dwell time in the range of 1-20 ms.

**Dwell time (ms)**	**1**	**4**	**10**	**16**	**20**
**Poisson, α=0.05, β=0.05**					
LOD (counts)	15.87	33.26	60.89	85.76	100.20
Particle count	71 ± 8.4	132 ± 11.5	91 ± 9.5	50 ± 7.1	56 ± 7.5
**Gaussian, σ=3.0**					
LOD (counts)	18.00	33.97	75.89	98.85	106.20
Particle count	63 ± 7.9	131 ± 11.4	48 ± 6.9	32 ± 5.7	43 ± 6.6

[1] Lockwood, T.E.; de Vega, R.G.; Clases, D. An interactive Python-based data processing platform for single particle and single cell ICP-MS. *J. Anal. At. Spectrom.* **2021**, *36*, 2536-2544. https://doi.org/10.1039/D1JA00297J

Table S4. Tested samples of cosmetic products.

№	Type	Commercial name	TiO_2 [a]	TiO_2NPs [a]
1	Face cream	Natura Siberica. Day face cream “Ukhod i uvlazhneniye (Care and moisturizing)” SPF 15	+	–
2	Foundation cream	Enough. Foundation cream “Collagen Moisture Foundation” SPF 15	+	–
3	Foundation cream	Avon. Mattifying foundation “Spokoynoye siyaniye (Calm glow)” SPF 20	+	+
4	Sunscreen lotion	Nivea. Mini sunscreen lotion “Zashchita i uvlazhneniye (Protection and hydration)” SPF 30	+	+
5	Face cream	Mary Kay. CC face cream SPF 15	+	+
6	Face cream	Garnier. Anti-aging face cream “Volshebnyy ukhod (Magic care)” SPF 20	– [b]	–

[a] according to declared composition

[b] possible trace concentration

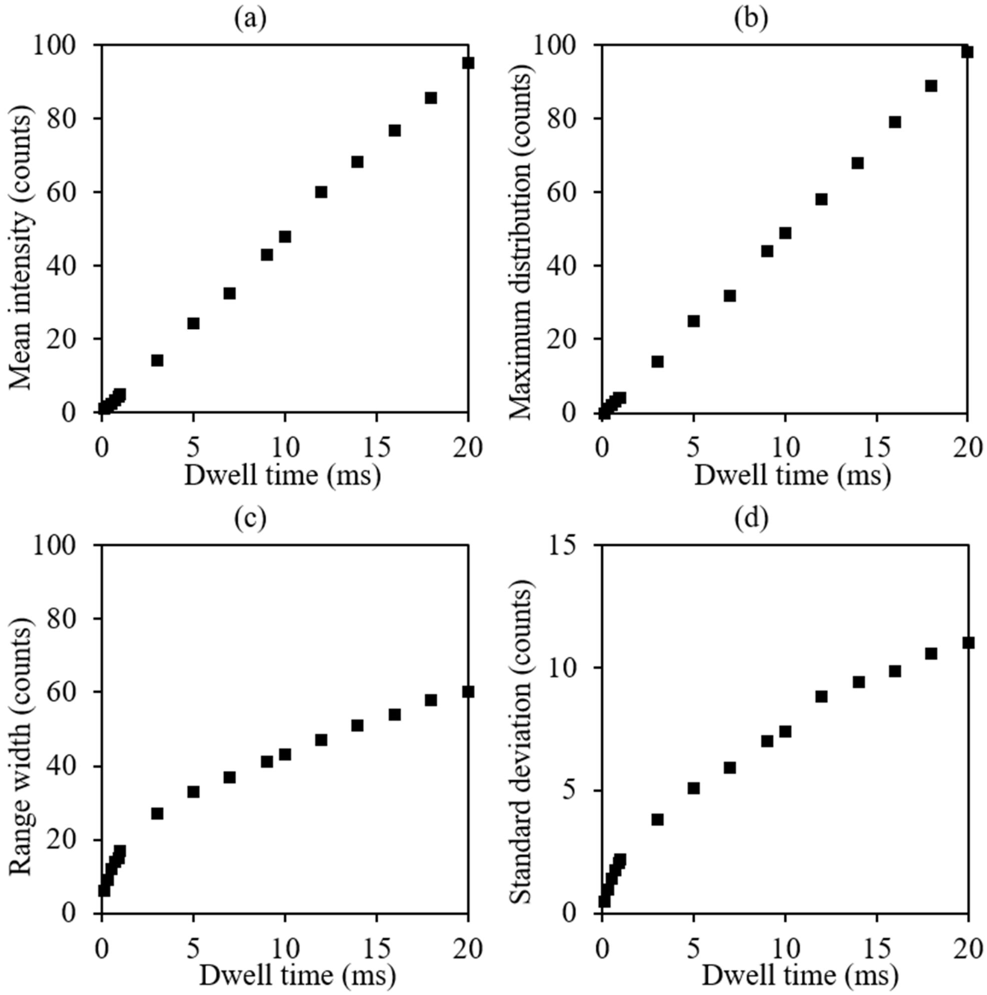

Figure S1. Changes in **(a)** the mean background intensity, **(b)** position of the distribution maximum on the intensity axis, **(c)** width of the oscillation range and **(d)** standard deviation of the background signal intensity in the dwell time range of 0.1-20 ms.

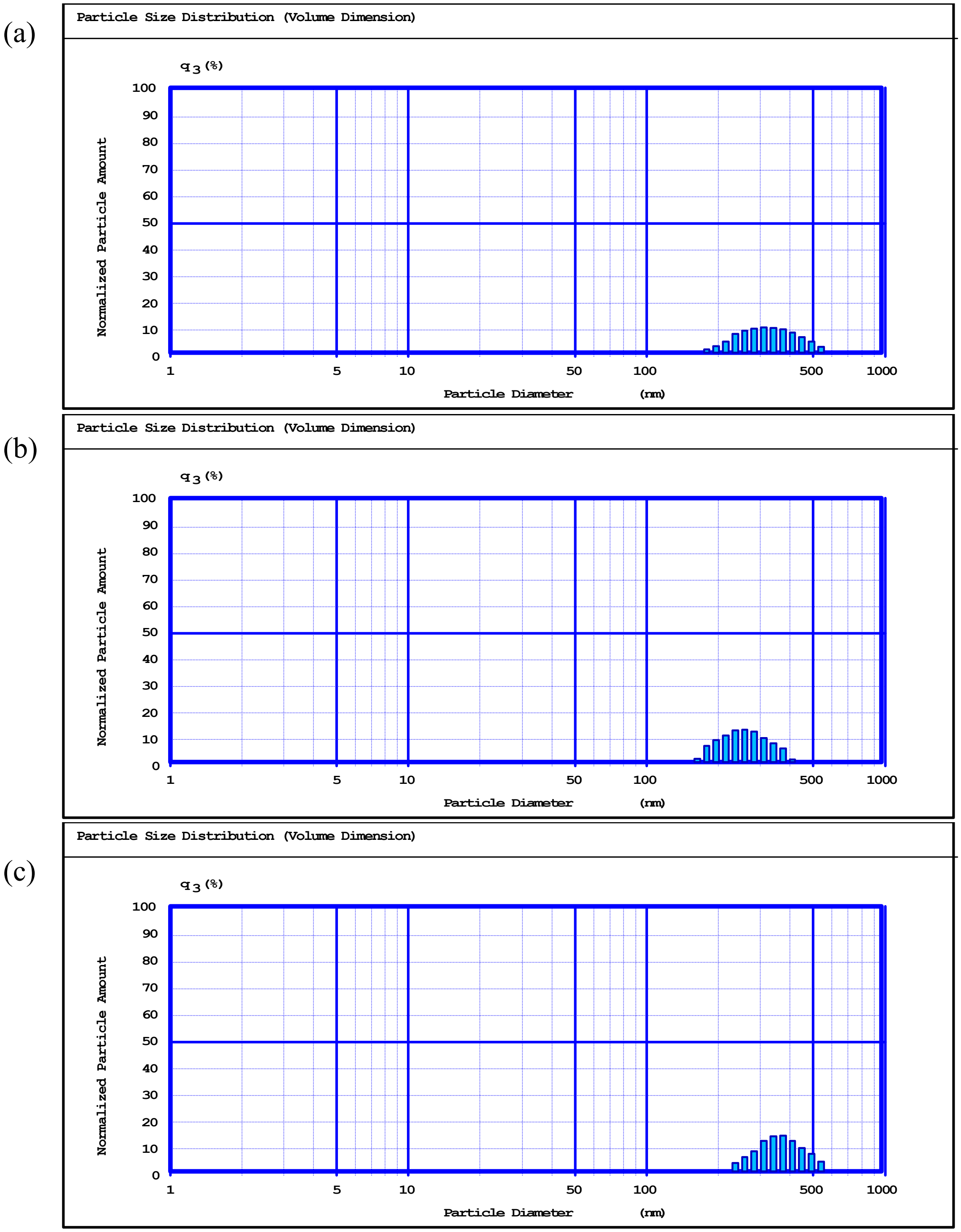

Figure S2. Particle size distributions (hydrodynamic diameter) for cosmetic product samples **(a)** 3, **(b)** 4 and **(c)** 5 (IG-1000 nanoparticle size analyzer (Shimadzu, Japan), voltage – 35 Vpp, frequency – 500 kHz, time – 0.10 sec).

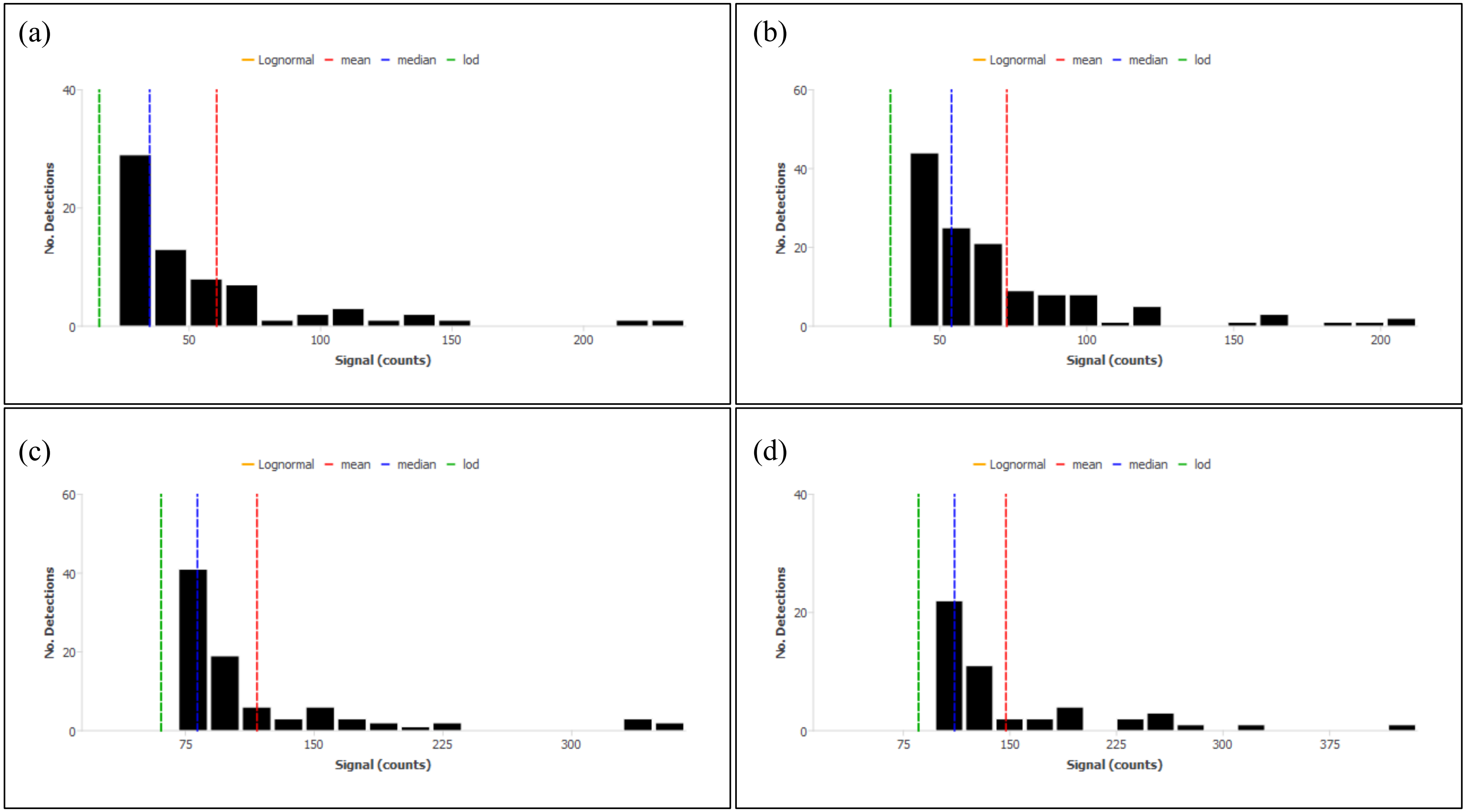

Figure S3. TiO_2 NP signal distributions in sample 3 at dwell times of (a) 1, (b) 4, (c) 10, and (d) 16 ms using SPCal software [1]

[1] Lockwood, T.E.; de Vega, R.G.; Clases, D. An interactive Python-based data processing platform for single particle and single cell ICP-MS. *J. Anal. At. Spectrom.* **2021**, *36*, 2536-2544. https://doi.org/10.1039/D1JA00297J.

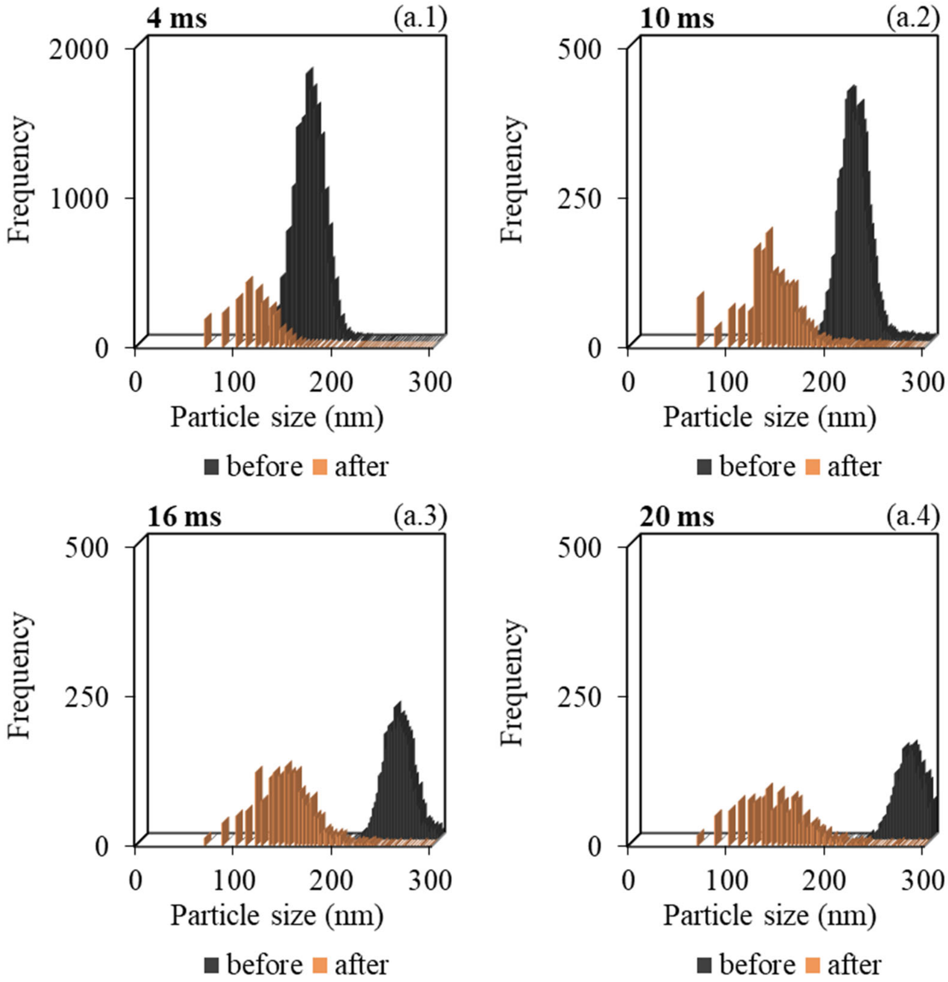

Figure S4a. TiO_2 NP size distribution before and after background signal correction at dwell times **(1)** 4, **(2)** 10, **(3)** 16 and **(4)** 20 ms in samples **(a)** 3, **(b)** 4, **(c)** 5 and 6 **(d, control)**.

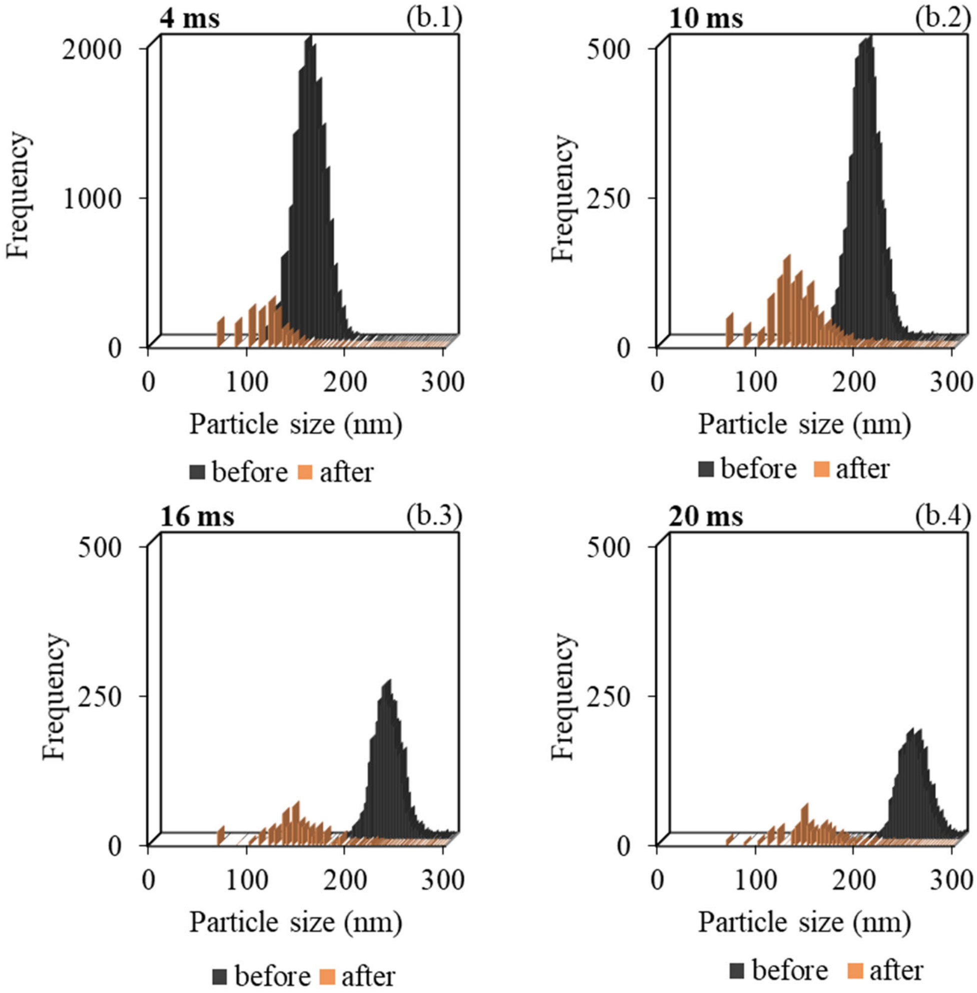

Figure S4b. TiO_2 NP size distribution before and after background signal correction at dwell times **(1)** 4, **(2)** 10, **(3)** 16 and **(4)** 20 ms in samples **(a)** 3, **(b)** 4, **(c)** 5 and 6 **(d, control)**.

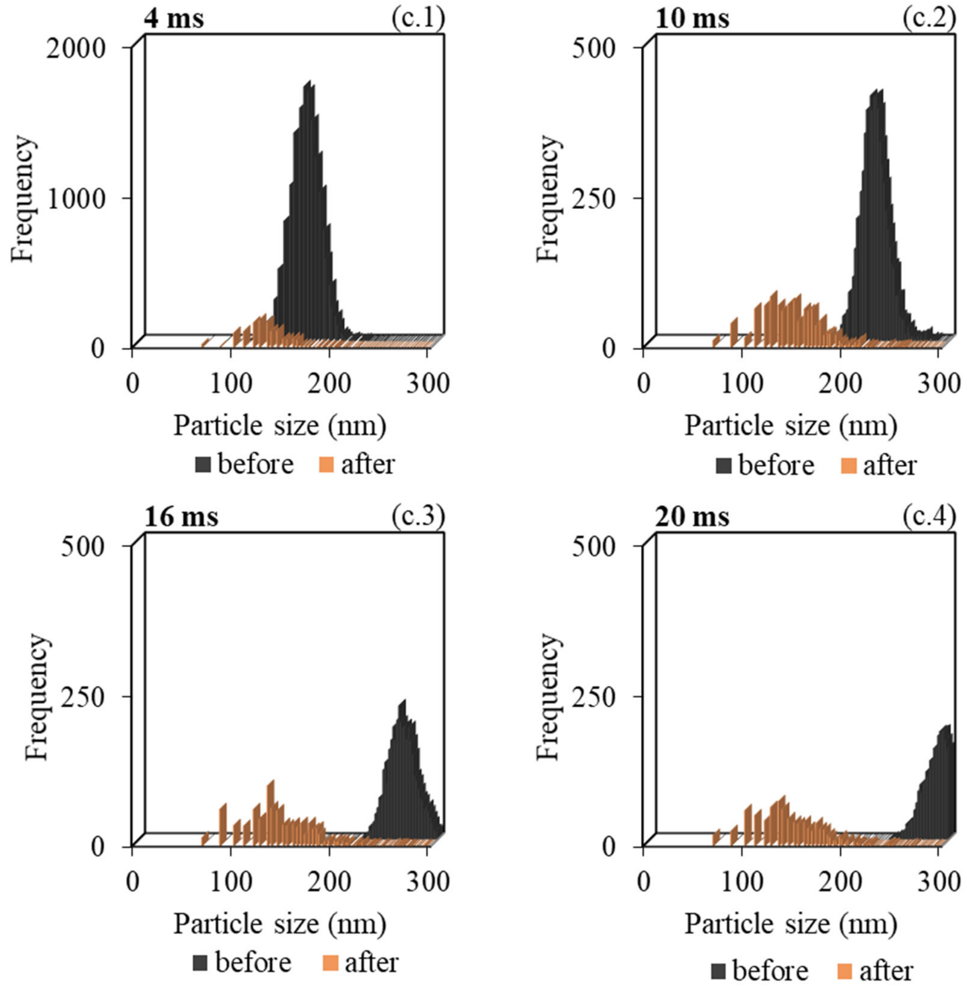

Figure S4c. TiO_2 NP size distribution before and after background signal correction at dwell times **(1)** 4, **(2)** 10, **(3)** 16 and **(4)** 20 ms in samples **(a)** 3, **(b)** 4, **(c)** 5 and 6 **(d, control)**.

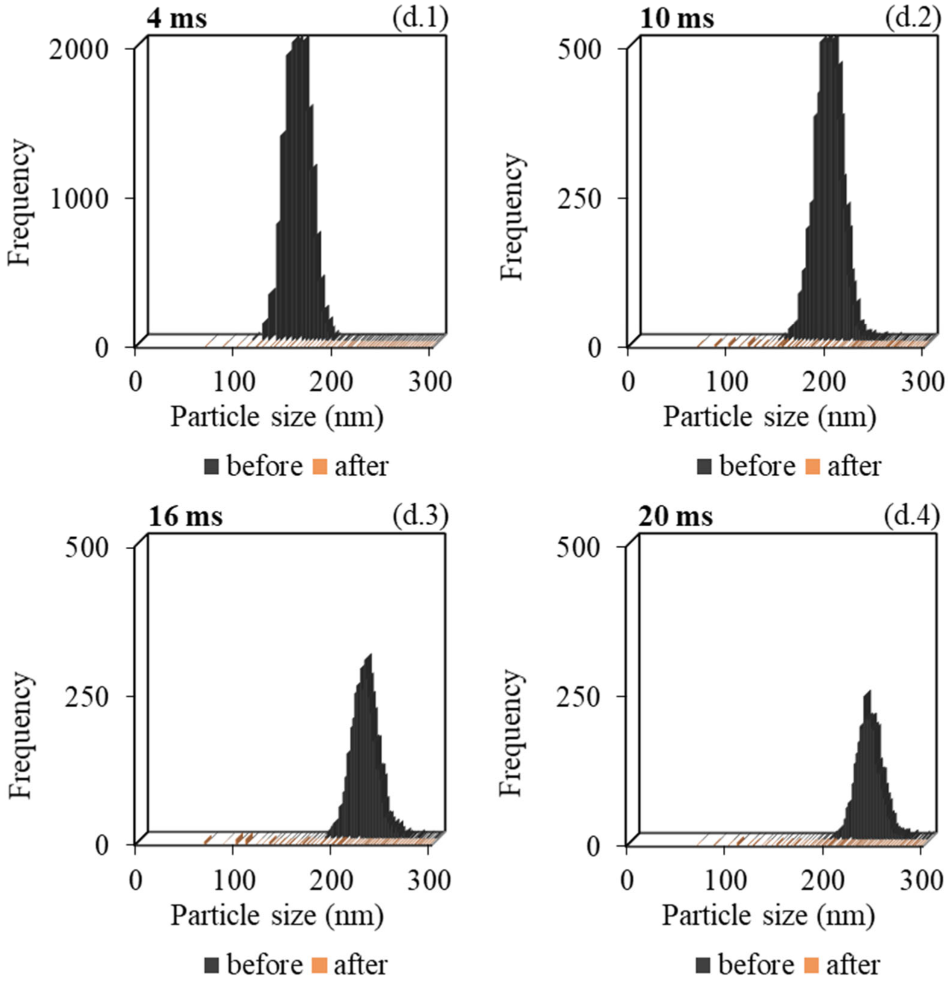

Figure S4d. TiO_2 NP size distribution before and after background signal correction at dwell times **(1)** 4, **(2)** 10, **(3)** 16 and **(4)** 20 ms in samples **(a)** 3, **(b)** 4, **(c)** 5 and 6 **(d, control)**.

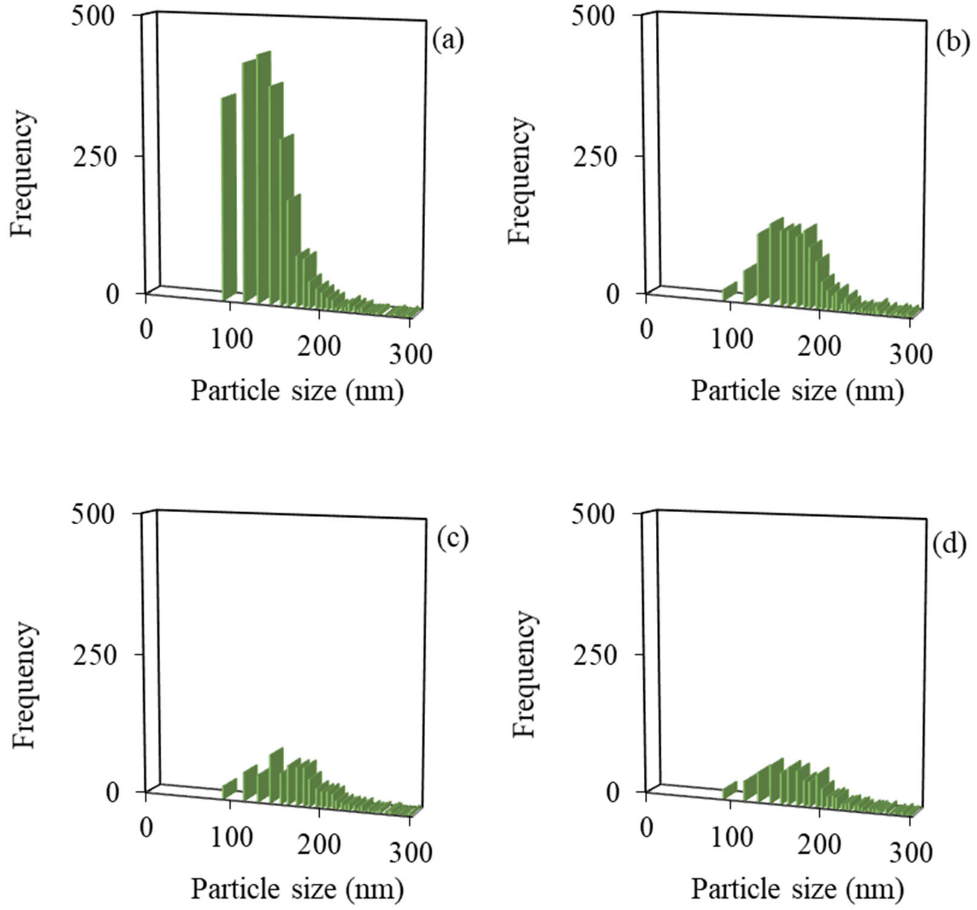

Figure S5. TiO_2 NP size distribution of sample 3 after background signal correction under optimized conditions at dwell times of **(a)** 4, **(b)** 10, **(c)** 16 and **(d)** 20 ms.
